# Empirical use of anakinra in AA amyloidosis of uncertain aetiology

**DOI:** 10.1186/1546-0096-13-S1-O70

**Published:** 2015-09-28

**Authors:** T Lane, DM Rowczenio, JA Gilbertson, JD Gillmore, AD Wechalekar, PN Hawkins, HJ Lachmann

**Affiliations:** 1 University College London, National Amyloidosis Centre, Division of Medicine, London, UK

## Introduction

AA amyloidosis is a serious complication of uncontrolled inflammation, which if left untreated will progress to renal failure and death. Effective suppression of the underlying inflammatory condition can halt organ damage or even lead to improved organ function. However, in 7% of our cohort the underlying inflammatory disease remains uncharacterised, creating a dilemma as to the choice of empirical treatment.

## Objectives

We empirically treated a small cohort of seven patients with AA amyloidosis of uncertain cause with the IL-1 receptor antagonist anakinra.

Patients and Methods: All seven patients were under the care of the UK National Amyloidosis Centre. Each patient underwent extensive investigation without diagnosing of the underlying inflammatory condition. Each patient subsequently underwent a trial of treatment with anakinra. Serum SAA and renal function as well as urine protein excretion were monitored closely, and all patients underwent serial SAP scintigraphy to monitor organ amyloid load.

## Results

Six of seven patients experienced suppression of inflammatory disease activity with the median pooled pre-anakinra SAA level falling from 63 mg/L (interquartile range, IQR, 42 - 119) to 5 mg/L (IQR 4 - 7). In these six patients this effect lasted for a median of 5.6 years, the duration of therapy, (IQR 2.4 - 7.6). In 2 patients proteinuria improved from 10.5 to 1.9 g/24 hr and 2 to 0.6 g/24 hr. Four patients showed regression of amyloid deposits on SAP scintigraphy. Five patients reported improvement in symptoms and one had been asymptomatic. One patient experienced no improvement in either inflammatory markers or in symptoms, and treatment with anakinra was discontinued.

## Conclusion

AA amyloidosis is a potentially reversible cause of renal failure. A therapeutic trial of anakinra is worth trying as it is potentially completely effective and has a better safety profile than high dose corticosteroids, other anti-cytokine or immunosuppressive drugs.

